# Effects of a Single Session of Mindfulness and Compassion on Skin Temperature in Breast Cancer Survivors

**DOI:** 10.3390/ijerph21081064

**Published:** 2024-08-14

**Authors:** David A. Rodríguez, Nadia Martínez, Li Erandi Tepepa Flores, Benjamín Domínguez, Patricia Cortés, Ana L. Chávez

**Affiliations:** 1 Department of Sociology, Division of Social Sciences and Humanities, Universidad Autónoma Metropolitana, Unidad Iztapalapa, Mexico City 09310, Mexico; analauchavez09@gmail.com; 2 Research and Graduate Studies Division, Faculty of Psychology, Universidad Nacional Autónoma de México, Mexico City 04510, Mexico; nadiamartinezcuervo@gmail.com (N.M.); benjamin@unam.mx (B.D.); 3 Centro de Atención y Evaluación Psicológica “Dr. Benjamín Domínguez”, Texcoco 56100, Mexico; li_erandi@hotmail.com; 4 Oncology Service, Centro Médico Nacional 20 de Noviembre, Instituto de Seguridad y Servicios Sociales de los Trabajadores del Estado, Mexico City 03229, Mexico; dra.pcortes@gmail.com

**Keywords:** skin temperature, mindfulness, compassion, affect, stress

## Abstract

Previous studies have suggested that mindfulness programs can be useful, in a significant sector of the population, to reduce stress when practiced for at least 8 weeks. The objective of the present investigation was to explore the effect of a single session of mindfulness practice in reducing stress in female cancer survivors. Two repeated measures studies were applied; in the first one, it was performed individually, while in the second one, it was performed in a group. Psychosocial measures were administered, and skin temperature was recorded as a marker of autonomic nervous activity. The results indicate that only when the mindfulness exercise was presented did the skin temperature increase (*p* < 0.05), with a large effect size (d > 0.8) during compassion, suggesting sympathetic decline. Furthermore, the psychosocial functioning of the group of female cancer survivors was like that of the non-clinical population. The data are discussed in the context of Polyvagal Theory, a theoretical model of biopsychosocial functioning, and evidence is provided on the effect of mindfulness and compassion on reducing stress and inducing positive affect in female cancer survivors.

## 1. Introduction

One of the priority populations with the highest risk of experiencing stress and emotional distress (high negative affect, anxiety, and mood disorders) and difficulties in their social behavior are women with breast cancer [1,2]. Furthermore, the restrictions on mobility, social interaction, and the reorganization of health services due to the COVID-19 pandemic (considered a public health emergency worldwide) led to evidence of deterioration in the physical and mental health of those who have suffered from the disease, experiencing symptoms of anxiety, depression, post-traumatic stress disorder (PTSD), cognitive alterations (attention and memory), and/or sleep disorders [3,4].

To reduce emotional distress, psychological strategies such as psychoeducation, cognitive-behavioral therapy, counseling, support groups, acceptance and commitment therapy, and mindfulness-based interventions have been proposed [5]. The latter has been developed not only to counteract emotional distress derived from COVID-19, but also to promote well-being through acceptance, and in cancer patients, their psychosocial effects during the COVID-19 pandemic have been reported [6,7].

MBI derives from the Buddhist roots of the practice of Mindfulness, which are characterized by focusing (anchoring) attention in the present moment, on a purpose (an object, activity, or situation), with openness, curiosity, and without judging the experience [8,9].

On the one hand, there is growing empirical evidence that demonstrates the effectiveness of MBI in mindfulness, to relieve psychological stress, and to reduce symptoms of anxiety, depression, and insomnia in women with breast cancer. By regulating attention they increase body awareness and promote physical, mental, and social well-being, which promotes acceptance and patience during the disease [10].

Although most of the aforementioned studies did not include Latino participants, there are studies in Spanish-speaking and Latino oncology populations with similar results that recognize the usefulness of mindfulness practice in improving psychological well-being. Elimimian, et al. [11] implemented an 8-week mindfulness-based stress reduction program in Spanish-speaking breast cancer survivors and found a significant reduction in symptoms of generalized anxiety, depression, and quality of life during 24 months of follow-up.

In Mexico, two studies reported the clinical effects of mindfulness on reducing pain and stress in the oncology population. In the first, García-Reyes et al. [12] applied a program of six individual mindfulness sessions in Mexican women with breast cancer, showing improvement in pain experience with a large effect size, although they reported a superior effect with the behavioral activation technique. Meanwhile, in the second, Gómez [13] conducted a group intervention based on mindfulness, acceptance and commitment, and assertiveness in Mexican women with breast cancer. The results showed significant changes in the reduction of perceived stress, an increase of emotions such as joy, and a reduction of anger, fear, and guilt.

On the other hand, the MBI with the greatest evidence in reducing negative affect, anxiety, and stress, reducing self-criticism, and increasing the feeling of well-being in general in populations with chronic diseases such as cancer and survivors of the same disease, is self-compassionate mindfulness [8,14,15,16,17]. Compassion can be defined as the desire to alleviate and avoid the suffering of oneself and others [9,18], whose practice provides tools to face uncertainty, setbacks, and failure [19].

When mindfulness based on compassion is practiced in a group, there is a greater probability of increasing prosocial behavior, the effect of which can spread ecologically to the immediate social environment and communities, improving the ability to infer negative mental states of other people and the ability to think and monitor one’s cognitive processes (metacognition) [20].

Most research in the field of mindfulness uses standardized psychometric measures to evaluate the effectiveness of intervention programs, the most common of which are useful to evaluate anxiety and depression, self-esteem, quality of life, fatigue, quality of sleep, and the ability to pay full attention [8,21,22,23,24]. In contrast to studies employing psychometric measures, few investigations employ physiological measurements to assess the short- and long-term effect of mindfulness-based psychological techniques in the oncology population; some studies report promising results on immune, endocrine, and autonomic responses [5,25,26,27,28].

At the level of the central nervous system, the main neurocognitive mechanisms of mindfulness have been identified, which suggest that focusing attention and consciously reducing value judgments to accept the emotional experience of the present moment influence the control and sustainability of attention which stimulates the anterior cingulate cortex, a structure with an important role in regulating emotional response [25]. The anterior cingulate cortex and the anterior insula (responsible for encoding bodily states) are activated when faced with the pain of others and when behavior is organized (either in plans or in desires to alleviate suffering) that together with the activation of the nucleus accumbens (involved in the reward circuit), mean that well-being is experienced, which gives rise to a compassionate response [20]. In other words, identifying suffering in oneself or another person and thinking about how to reduce it can generate a feeling of engagement.

At the level of the peripheral nervous system, the Polyvagal Theory [29] proposes that a positive affective state of low physiological arousal, such as those experienced with the practice of MBIs, promotes feelings of security, calm, curiosity, and well-being; additionally, it improves attention control and facilitates social connection, mainly compassionate response; meanwhile, the defense response of the cortico-limbic pathways is inhibited, which increases the desire to alleviate the suffering of oneself and others [30]. This affective state implies a sympathetic tone of low activation, which can be evaluated through peripheral psychophysiological recordings (non-invasive and painless measures for patients diagnosed with chronic oncological disease). Consequently, some studies have been interested not only in the psychometric assessment of the affective state but also in the dynamic psychophysiological evaluation of the effects of mindfulness practice on neurovegetative responses [23,26,27,28].

One of the measures to evaluate sympathetic tone is skin temperature, which can be recorded using infrared light thermometers or infrared thermal imaging. Skin temperature responds to emotional changes (from one emotional state to another); for example, stressful psychosocial situations, such as the social isolation that was experienced from one moment to the next during the confinement due to the pandemic, generated emotional discomfort, fear, anxiety, pain, and depression, inducing a sympathetic vasoconstriction reflex, particularly in the distal regions of the body (hands, feet, nasal tip, ears). When a blood vessel is constricted, blood volume is less in that region, which decreases skin temperature. On the contrary, if the person experiences the positive affect of low physiological arousal, such as calm, interest, and curiosity, the blood vessels dilate and increase the temperature in these same regions [31,32].

Frequently, MBI usually lasts between 6 and 8 weeks with group sessions of 45–120 min that include psychoeducation, mindfulness practice, group discussions, and home exercises [10,27,33,34,35]. There are few reports of the practice of mindfulness in a single session, the main results of which indicate that a session of 30 min increases skin temperature [36,37]; a session of 25 min reduces psychological tension, confusion, feelings of anguish, anger, and fatigue [38]; 20 min of mindfulness reduce the galvanic response of the skin [39]; 10 min of practice [40] improve executive attention; 5 min reduce suffering [41] and stress levels and improve the feeling of calm and relaxation [42].

Some studies have analyzed affective states on facial temperature through Infrared thermal image (IRT) in the oncology population [43]. Mindfulness practice has been reported to increase the temperature of the forehead area in expert meditators [37], while in novice meditators the temperature of the periorbital area rises [36]. However, the use of face masks during the COVID-19 pandemic limited the thermal recording of the tip of the nose, so the present research proposed to examine the psychophysiological effect of an MBI on the skin temperature of the hands.

Facial temperature is not the only region of interest that changes as a function of emotional arousal. The temperature of the hands (palms, back, and fingertips) is also sensitive to the autonomic activity of the emotional state, whose measurement and physiological regulation have antecedents in the practice of thermal biofeedback [44,45]. However, there is not enough evidence to allow us to understand the immediate effect of mindfulness-based techniques in the clinical oncology population on skin temperature in the hands.

Based on the studies reviewed, the following research question was proposed: What is the effect of a Mindfulness and Compassionate Mindfulness session on the peripheral temperature of the hands in female breast cancer survivors? The objectives of the present study were two: first, to examine the affective state and psychosocial functioning of a group of breast cancer survivors at two moments after confinement due to COVID-19; second, to explore the effect of the practice of a single session of mindfulness and a single session of compassion on peripheral skin temperature, in two modalities: individual (under conditions of social restriction) and group (in circumstances of return to face-to-face activities).

## 2. Materials and Methods

Two studies were conducted: the first, the individual application of a compassion and mindfulness protocol during October and November 2021, respectively, in a controlled environment to reduce environmental and social variables on the thermal psychophysiological response, in a social context of confinement in Mexico due to the COVID-19 pandemic; while in the second study, a similar protocol was applied, with different mindfulness and compassion techniques, in the patients’ natural environment during one of the group meetings held by the group of female cancer survivors once a month in the hospital facilities where they receive medical care, during March 2023, a post-confinement stage, to replicate the results of the first trial in an ecological environmental and psychosocial context. This means that Study 1 was carried out in the context of confinement, so participants had to practice mindfulness individually under controlled conditions. Once confinement was over, Study 2 sought to replicate the psychophysiological effects of the practice of mindfulness techniques in a natural context of a group meeting of these patients to examine whether the effect of a single session of mindfulness was also achieved if it was practiced in a group, which would reduce the application time of this intervention with greater collective scope.

### 2.1. Study 1. Individual Trial: Effect of Mindfulness and Compassionate Mindfulness on Skin Temperature

#### 2.1.1. Participants

Through non-probabilistic sampling, the Oncology service of the National Medical Center 20 de Noviembre-ISSSTE, in Mexico City, referred 16 female breast cancer survivors to participate in the individual trial with a mean age = 63.36 (±11.39) years. The nature of this study was exploratory, and even more so in the peri- and post-confinement (social isolation) context. For this research, the medical oncologist determined which cancer survivors might require psychosocial intervention to reduce distress. All participants met the criteria proposed by the guidelines of The American Academy of Thermology [46] for the thermographic assessment of intrinsic individual factors (fasting from food or drugs for around 8 h, free of skin cosmetics and without the presence of physical discomfort at the time of participating in the study) and extrinsic factors (they were scheduled between 10 a.m. and 12 p.m. in a large, bright, ventilated university room with temperature control of ≈22 °C). All participants completed their evaluations, so no patient was excluded or eliminated.

#### 2.1.2. Instruments and Materials

Patient Health Questionnaire (PHQ-4) [47]. A self-administered questionnaire assessed depressed mood and anxious symptoms. It consists of four items (two items for anxiety and two for depression) with a Likert-type response from 0 to 3. The total score was determined by adding together the scores of each of the four items. Scores were rated as normal (0–2), mild (3–5), moderate (6–8), and severe (9–12). A total score ≥3 for the first two questions suggested anxiety. A total score ≥3 for the last two questions suggested depression. It presented a validity of 84% of the explained variance of the two factors (anxiety and depression), with a reliability of 0.82 and 0.90 respectively.

Disposition to Receive Social Support Scale (EDaRAS) [48]. The study used a psychometric scale of seven items on a Likert-type scale with five response options on the willingness to receive social support, ranging from 1—Never to 5—Always. The test contained a single factor that explained 44.59% of the variance and was validated in the Mexican population with a good level of reliability (α = 0.791).

Positive Affect/Negative Affect Scale in Mexico (APAN-M) [49]. This consisted of 20 items on 10 positive and 10 negative affective states, with a Likert-type response scale of five response options (1—Never, 5—Always) and a good level of reliability for each affective valence (α = 0.88 and 0.92, respectively).

DM300 Infrared Digital Thermometer. A digital, portable, infrared light thermometer with an accuracy of ±0.2 °C was used to record the temperature without contact with the person’s skin, with a capture diameter of 0.5 cm^2^.

Infrared Thermal Imaging. Thermal images of the hands were acquired using a low-cost, low-resolution infrared thermal camera (80 × 60 pixels) [50], with progressive scan, FOV—Diagonal 63.5°, FOV—Horizontal 50° (nominal), Effective Frame Rate—8.6 Hz, Scene Dynamic Range—−10 to 140 °C (gain high); up to 450 °C (low gain), typical, Spectral range—Longwave infrared, 8 µm to 14 µm, Thermal sensitivity—<50 mK (0.050 °C). This is an easy-to-use portable thermal monitoring screening tool, whose clinical usefulness has been discussed in various health evaluation protocols [51,52]. The system captured one image per second at an ambient temperature of 20 to 22 °C and 20% humidity.

#### 2.1.3. Design and Procedure

**Figure 1:**
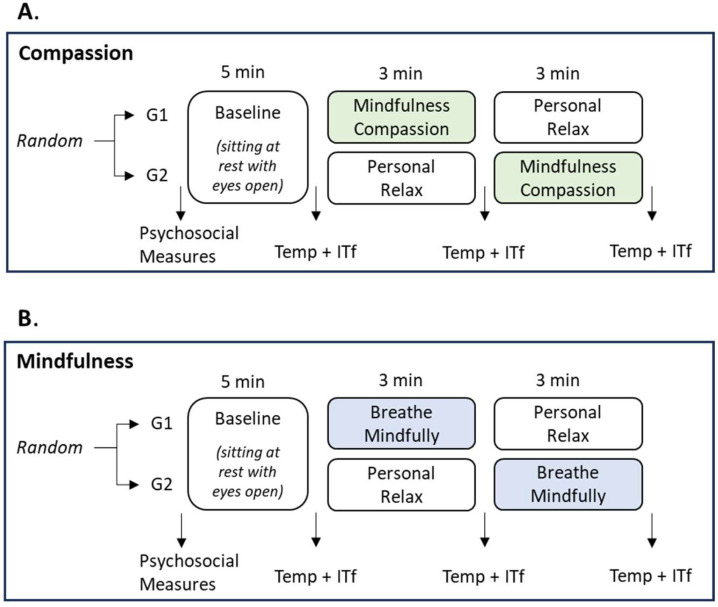
Research design. Note. Repeated Measures Crossover Design. The compassion design used is presented in (**A**), while the mindfulness breathing design is shown in (**B**). Temp = Skin Temperature, ITf = Infrared Thermal Imaging, G1 = Group 1, G2 = Group 2.

To assess the emotional and psychosocial state of the participants, a non-experimental, cross-sectional design was used; meanwhile, to evaluate the effect of mindfulness and compassionate mindfulness on skin temperature, a repeated measures crossover design was proposed (Figure 1). This design had the advantage of applying the same procedures to all participants but in a different order to estimate whether there was a carryover effect from one activity to another and that thermal variations were not explained by the passage of time to weigh the effect of the MBI on skin temperature.

Single-session compassionate mindfulness practice was performed in October 2021 (A), and the single-session effect of mindful breathing was examined in November 2021 (B).

For both the practice of Compassion (A) and Mindful Breathing (B) the same procedure was followed:

1. First, the participants were randomized to form two groups:

Group 1. Baseline, (B) compassionate mindfulness/(A) mindfulness and personal relaxation.

Group 2. Baseline, personal relaxation, and (B) compassionate mindfulness/(A) mindfulness.

2. Second, all participants signed their informed consent based on the Declaration of Helsinki, and subsequently the following psychosocial measures were applied: PHQ-4, Disposition to Receive Social Support Scale, and APAN-M, which allowed us to assess the emotional state and socio-affective functioning.

3. Third, they sat at rest for at least 5 min (baseline). At the end of this time, the initial temperature was recorded at the tip of the left middle finger.

4. Fourth, Group 1 received a brief (3 min) compassionate mindfulness exercise, while Group 2 was asked to relax as the patients knew how to do (personal relaxation) for 3 min. Subsequently, the tasks were reversed: those patients who began with personal relaxation followed the practice of compassionate full attention/mindfulness and vice versa.

The compassionate mindfulness exercise (Self-Compassion) was adapted from Tovar, et al. [53], whose indications are presented in Appendix A.

Meanwhile, the breathing exercise with mindfulness was adapted from the INCMNSZ Mental Health Brigade [54], whose indications are presented in Appendix B.

The instructions given to the patients for personal relaxation (without psychological support) were:

*Now I am going to ask you to take a couple of minutes to make yourself comfortable, calm, and collected. You can keep your eyes open or closed, however you prefer. I’ll let you know when we’re done.*

At the end of each activity (baseline, compassionate mindfulness, mindfulness, and personal relaxation) their skin temperature was recorded).

### 2.2. Study 2. The Practice of Mindfulness and Compassionate Mindfulness on Skin Temperature in a Single Group Session

#### 2.2.1. Participants

Through non-probabilistic sampling, 25 female breast cancer survivors with a mean age = 63.66 (±13.93) years, attended by the National Medical Center 20 de Noviembre-ISSSTE in Mexico City, and voluntarily participated. All participants met the criteria for thermographic assessment according to environmental temperature ≈ 20 °C, and intrinsic and extrinsic individual factors [46]. All participants completed their evaluations, so none of them were excluded or eliminated.

#### 2.2.2. Instruments

*Compassion Scale for Mexican population (ECOM)* [55]. The study used a self-applicable psychometric instrument, with 17 items, divided into three factors: (1) Motivation to alleviate suffering, (2) Affective reaction to suffering, and (3) Compassion towards animals, with response options on a Likert-type scale with seven response options, which ranged from “never” to “always”. The scale had a good level of reliability (α = 0.90) and validity (R^2^ = 53.59%).

Mindfulness Scale [56]. This was a self-report scale with 12 items, divided into two factors: (1) Attention, and (2) Acceptance, with response options on a Likert-type scale with five response options, ranging from “never” to “always”. The scale had a good level of reliability (α = 0.86) and validity (R^2^ = 56.99%).

DM300 Infrared Digital Thermometer. A portable handheld infrared light thermometer with an accuracy of ± 0.2 °C was used to record the temperature without contact with the person’s skin, with a capture diameter of 0.5 cm^2^.

Low-resolution Infrared Thermal Camera. Facial and hand thermal images were acquired using a low-cost, low-resolution infrared thermal camera [50]. It had a FLIR Lepton V2 thermographic sensor with a resolution of 80 × 60 pixels and a sensitivity of 0.05 °C. The system captured one image per second, at an ambient temperature of 22 °C and 26% humidity. The display to show visual images was 7 inches with a resolution of 800 × 480 pixels. Numeral 3 of the guideline proposed by The American Academy of Thermology [46] was considered.

Visual Analog Scale of Subjective Stress. This scale (0–10), where 0 is no perceived stress and 10 is maximum perceived stress, is useful for assessing subjective self-report of perceived stress levels before and after an intervention.

WordItOut [57] is a free program that allows the user to organize words. This program was useful to qualitatively represent the subjective self-report of the mindfulness-based practice. At the end of the practice, the researcher asked all participants to write down their positive and negative experiences on a sheet of paper; all words expressing emotional states were placed in the program to be organized based on their frequency. That is, the words that were repeated more often were larger and the words that were repeated less often were smaller.

#### 2.2.3. Design and Procedure

**Figure 2:**
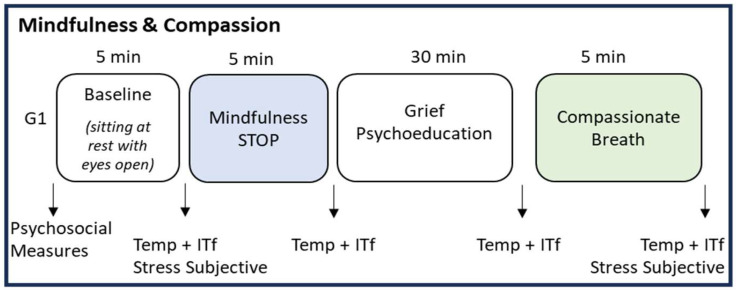
Research design. Note. Temp = Skin Temperature, ITf = Infrared Thermal Imaging, G1 = Unique Group.

For the group application of the single session of mindfulness, two designs were used: first, a cross-sectional observational design for the psychosocial measures, and second, a single-group repeated measures design for the thermographic evaluation (Figure 2).

First, the participants were summoned to their group session at noon in a lighted room at the Centro Médico Nacional 20 de Noviembre hospital, Mexico City, with natural ventilation at an ambient temperature of 24 °C. As they arrived, they took their seats and began to answer the psychosocial measures of mindfulness and compassion. Afterwards, they remained seated for a few minutes to complete acclimatization to room temperature. Before starting the initial skin temperature recording, they were asked using a visual analog stress scale how much stress they perceived on a scale of 0–10, where 0 was no stress and 10 was the maximum perceived stress.

Second, a psychologist external to the study applied a mindfulness exercise, lasting 5 min, called STOP (Stop, Take a breath, Observe, and Proceed), adapted from the INCMNSZ Mental Health Brigade [54], whose indications are presented in Appendix C. At the end of the mindfulness exercise, skin temperature was recorded again.

Third, a psychoeducational talk on grief was given, as part of their group activities that they normally carry out in their periodic meetings. After this talk, the skin temperature was recorded again.

Fourth, a second psychologist trained in mindfulness applied a compassionate mindfulness exercise (Compassionate Breathing & Feeling Safe with Your Benefactor), adapted from Collard [58], whose indications are presented in Appendix D, and at the end the skin temperature was recorded for the last time in line with the visual analog stress scale (0–10).

Finally, the participants were asked to write freely on a sheet of paper about their affective experience of these exercises or if they had difficulty following the instructions for mindfulness and compassionate mindfulness. This last activity served to analyze affective language, and whether it was consistent with the temperature changes associated with the effects of mindfulness and compassionate mindfulness.

## 3. Results

### 3.1. Study 1. Individual Mindfulness and Compassion on Skin Temperature

#### 3.1.1. Mental Health and Psychosocial Measures

The psychological evaluation before the Mindfulness practice revealed that no patient presented anxious or depressive symptoms (anxiety mean = 1.25 ± 0.17; depression mean = 0.56 ± 0.18).

**Figure 3:**
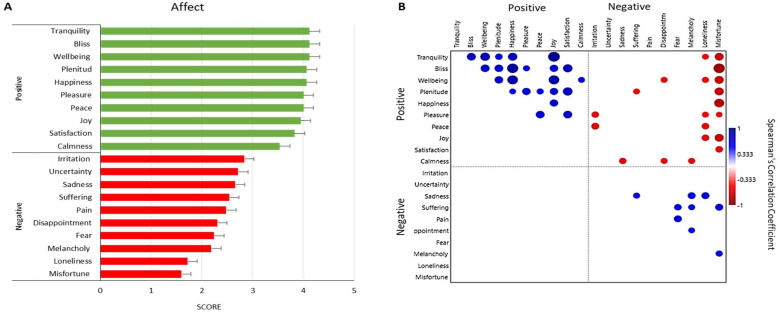
Affective states and their correlation in the group of breast cancer survivors. Note. In (**A**) it is observed that the group presented a predominance in the positive affect score. In (**B**) the correlation of significant associations (*p* < 0.05) between positive affects, positive and negative affects, and correlations between negative affects is shown. Positive correlations appear in blue (between 0.33 to 0.77), while negative correlations appear in red (−0.33 to −0.73).

In turn, the analysis of the Positive Affect/Negative Affect Scale score in Mexico revealed a positive affective balance (positive mean = 3.98 ± 0.92; and negative mean = 2.32 ± 0.90), which is consistent with low or null anxious or depressive symptoms. The affections with the highest scores were: tranquility, calm, and well-being; while the least reported were: loneliness and misfortune. Figure 3A shows the mean and standard error of the scores for each affective state, while Figure 3B shows the significant correlations (*p* < 0.05) between positive and negative affections, whose overall scores presented an inverse association (rho = −0.77, *p* < 0.001). For its part, the anxiety score correlated inversely with the happiness score (rho = −0.54, *p* = 0.029), and directly with the misery score (rho = −0.63, *p* = 0.009), while the depression score was directly associated with melancholy (rho = 0.63, *p* = 0.034) and unhappiness (rho = 0.54, *p* = 0.007); and inversely with the scores of tranquility (rho = −0.52, *p* = 0.040), happiness (rho = −0.69, *p* = 0.002), well-being (rho = −0.54, *p* = 0.030), joy (rho = −0.73, *p* = 0.001), and satisfaction (rho = −0.59, *p* = 0.016), and generally with the positive affect score (rho = −0.65, *p* = 0.006). Furthermore, three positive affective states showed a negative correlation with systolic blood pressure: calmness (rho = −0.62, *p* = 0.01), well-being (rho = −0.64, *p* = 0.030), and joy (rho = −0.68, *p* = 0.003).

On the other hand, the score of the Disposition to Receive Social Support Scale was M = 3.66 ± 0.21, it did not differ from the non-clinical population reported by Sánchez & Calleja [48] M = 3.57 ± 0.74 (t = 0.46, (15), *p* > 0.05, d = 0.11). No significant associations (*p* > 0.05) were found with any other measure; only the score of item 1 (I share my feelings with other people to see if they help me) exhibited a positive correlation with the level of happiness (rho = 0.51, *p* = 0.045).

#### 3.1.2. Thermal Effects of a Single Session of Compassionate Mindfulness and a Single Session of Mindful Breathing

**Figure 4:**
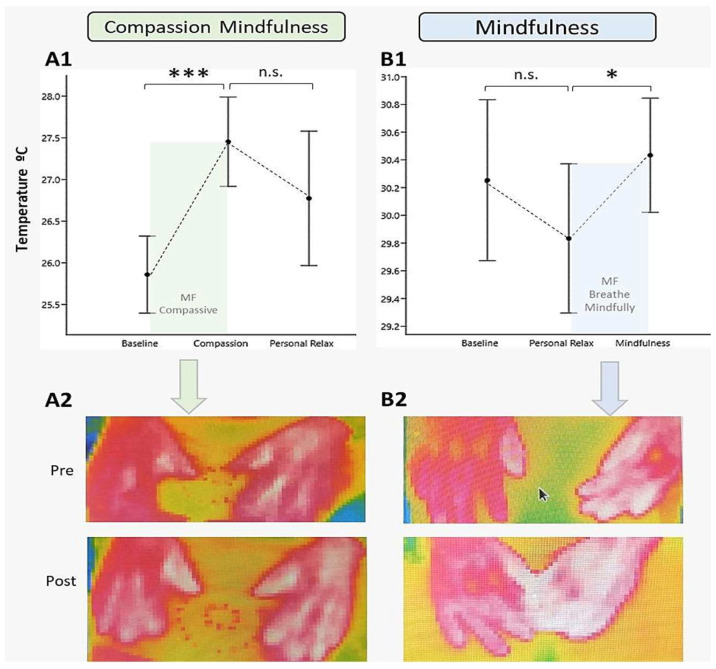
Effect of Compassionate Mindfulness and Mindfulness on skin temperature in an individual session. Note. In (**A1**) the effect of compassionate mindfulness on skin temperature is presented, while in (**A2**) the infrared thermal image of the hands before and after this exercise is shown. In (**B1**) the effect of mindful breathing appears and in (**B2**) the infrared thermal image of the pre-post-exercise skin temperature is observed. The mean and standard error of skin temperature are represented in (**A1**,**B1**). MF = Mindfulness. * = *p* < 0.05, *** = *p* < 0.001, n.s. = not significant.

The compassionate mindfulness session elicited significant changes in skin temperature (F (1.45, 20.33) = 4.66, *p* = 0.031, η^2^ = 0.074). In particular, temperature increased significantly when the compassionate mindfulness exercise was applied with a large effect size (from baseline M = 25.9 °C ± 1.79 °C, to compassion Mean = 27.5 °C ± 2.07 °C, t = 4.54, (15), *p* < 0.001, d = 1.17, [CI: 95% 0.49, 1.82]); and subsequently decreased slightly but not significantly when the patients were asked to relax on their own (baseline M = 26.8 °C ± 3.12 °C, t = −1.27, (15), *p* = 0.22, d = −0.33, [CI: 95% −0.84, 0.19]) (Figure 4A1).

No significant difference was found between baseline temperature and personal relaxation (t = 1.42, (15), *p* = 0.17, d = 0.36, [CI 95%: −0.16, 0.88]). The order of task presentation did not have a thermal carryover effect; that is, no significant differences were found between the two subgroups: those who began with the practice of compassionate mindfulness (baseline–compassionate mindfulness–personal relaxation) and those who began with personal relaxation (baseline–personal relaxation–compassionate mindfulness), in none of the three measurements: baseline (U = 20, *p* = 0.397, r = 0.285); compassionate mindfulness (U = 26, *p* = 0.862, r = 0.0714); personal relaxation (U = 25, *p* = 0.772, r = 0.107), so the main effect is solely due to compassionate mindfulness practice.

In the single session of mindfulness, a similar result was observed in skin temperature between personal relaxation and baseline (t = −0.94, (14), *p* = 0.36, d = −0.24 [CI 95%: −0.75, 0.27]); meanwhile, with mindful breathing the temperature increased moderately (t = 2.21, (14), *p* = 0.044, d = 0.57 [CI 95%: 0.015, 1.11]) (Figure 4B1). The infrared thermal image shows the pre-post exercise effect of Compassion (Figure 4A2) and pre-post Breathing (Figure 4B2) with Mindfulness on the skin temperature of the back and palm of the hands.

### 3.2. Study 2. Psychosocial Measures and Skin Temperature

#### 3.2.1. Mindfulness

The psychometric mindfulness scale revealed that the mean attention score of the oncology group (M = 3.09 ± 0.75) was not different from the mean of the reference group of the scale described by the author in a non-clinical population (M = 3.14 ± 0.60) (t = −0.293, (24), *p* = 0.77, d = −0.067). There were also no significant differences in the acceptance score between the group of female cancer survivors (M = 3.90 ± 0.90) and the group of young adults (M = 3.82 ± 0.67) (t = 0.40, (24), *p* = 0.69, d = 0.092). The psychometric score of attention was positively and moderately correlated with compassion towards animals (rho = 0.50, *p* = 0.029).

#### 3.2.2. Compassion

The assessment of the Compassion Scale for the Mexican population indicated that the M = 5.35 ± 0.80 of the oncology group was like that reported by the authors López & Moreno-Coutiño [55] in the general population (M = 5.42 ± 0.84) (t = −0.363, (24), *p* = 0.721 d = −0.082). However, in the analysis of the subscales, the level of Motivation to Relieve Suffering of female breast cancer survivors M = 5.42 ± 0.82 was placed moderately above that of the general population (M = 4.99 ± 0.87) (t = 2.29 (24), *p* = 0.034, d = 0.52); their Affective Reaction to Suffering score M = 5.58 ± 1.19 was similar to that of the general population (M = 5.74 ± 1.13) (t = −0.59, *p* > 0.05, d = −0.135); while the Compassion towards animals score (M = 4.82 ± 1.26) was moderately lower compared to the reference group (M = 5.51 ± 1.19) (t = −2.36, (24), *p* = 0.030, d = −0.54). Furthermore, compassion towards animals showed a correlation with skin temperature (rho = 0.47, *p* = 0.042).

#### 3.2.3. Stress

**Figure 5:**
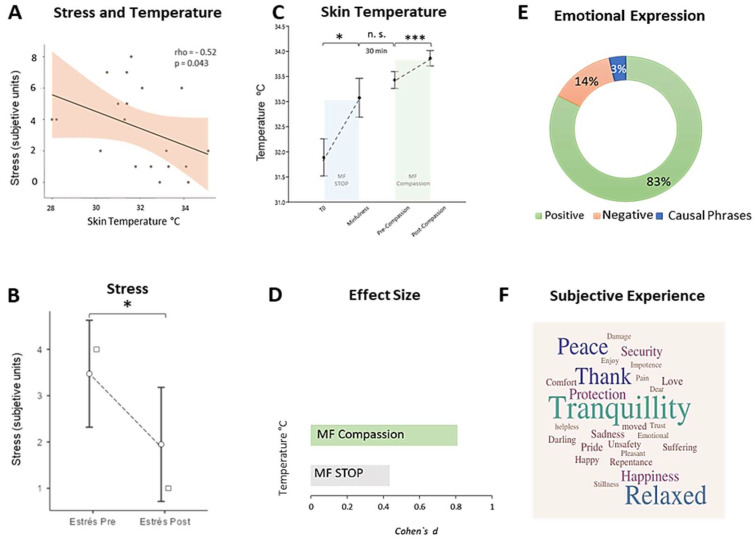
Effect of Mindfulness and Compassionate Mindfulness group practice on skin temperature, subjective stress score, and affective language. Note. In (**A**) the correlation between skin temperature and the subjective stress unit score is presented. In (**B**) the effect of a pre-post single session of mindfulness and compassionate mindfulness on the subjective stress score is shown, while in (**C**) the effect on skin temperature appears (in the interval between an exercise and another, no significant differences were found, so the thermal increase of the skin only occurred when the care techniques were performed). In (**D**) Cohen’s d effect sizes on skin temperature were calculated. In (**E**) the percentage relationship of the affective language that the patients expressed during compassionate mindfulness is presented, while in (**F**) the affects (positive and negative) that they experienced in this technique are highlighted. MF STOP = Mindfulness Stop, Breathe, Observe and Proceed, Take a breath, Observe and Proceed. * = *p* < 0.05, *** = *p* < 0.001, n.s. = not significant.

The subjective stress score was negatively associated with baseline skin temperature (rho = −0.53, *p* = 0.043) (Figure 5A). After the mindfulness group session, the perception of stress significantly decreased (W = 31.5, *p* = 0.034, r = −0.588) from a Mdn pre = 4 units of subjective stress to a Mdn post = 1 (Figure 5B).

### 3.3. Core and Skin Temperature

The recording of core temperature indicated that no patient presented fever (M = 36.2 °C ± 0.83, [95% CI 35.9, 36.6]), whose distribution was not normal (*p* < 0.001), (Mdn = 36.6 °C).

Single-session mindfulness and compassion practice induced a significant increase in skin temperature (X^2^ = 34.9, (3), *p* < 0.001) (Figure 5C). The practice of mindfulness exercise significantly increased skin temperature (M pre = 31.9 °C ± 1.84 °C to M post = 33.1°C ± 1.93 °C), (W = 223, *p* = 0.038, r = 0.487). Subsequently, during the 30 min of psychoeducational talk, skin temperature was maintained (W = 203, *p* > 0.05). Finally, the application of compassionate mindfulness significantly increased peripheral temperature from M pre = 33.4 °C ± 0.84 °C to M post = 33.9 ± 0.75 °C, with a large effect size (t = 3.94, (24), *p* < 0.001, d = 0.806, [CI 95%: 0.33, 1.26]) (Figure 5D).

Additionally, data analysis revealed that skin temperature remained below 34 °C in the session during the pre-mindfulness measurement (t = −5.73), (24), *p* < 0.001, d = −1.14); and pre-compassionate mindfulness (t = −3.23, (24), *p* = 0.004, d = −0.648). However, at the end of the compassionate mindfulness practice, a deep state of tranquility was reached (t = −0.89, (24), *p* = 0.37, d = −0.18).

### 3.4. Affection

When examining the subjective experience of compassionate mindfulness practice, the analysis of affective language revealed a predominance of positive affect, in more than 80% of the written words (Figure 5E), the contents of which revealed experiencing deep tranquility, gratitude, relaxation, and peace, mainly (Figure 5F).

### 3.5. Comparison of Skin Temperature between Female Cancer Survivors and Women without Disease

The results found in Study 1 are comparable with the resting temperature data of the non-clinical population by Reséndiz, et al. [45], who reported a left middle fingertip temperature M = 30.66 °C. During the single-session practice of compassionate mindfulness in study 1, in the breast cancer survivors the temperature of this region was below the average of the non-clinical population at pre-compassion rest (*p* < 0.001, d = −2.68), post-compassion (*p* < 0.001, d = −1.54), and ending personal relaxation (*p* < 0.001, d = −1.24). However, for the single session of mindful breathing, the left finger temperature of cancer survivor patients reached a similar temperature to that of women without disease during rest (*p* = 0.495), personal relaxation (*p* = 0.146), and the practice of mindfulness (*p* = 0.591). In contrast, post-mindful breathing skin temperature (in the cancer survivor group) was significantly higher compared to the social stress response (*p* = 0.048, d = 0.56) in disease-free women.

In Study 2, during single-session group practice of mindfulness and compassionate mindfulness, the resting skin temperature of breast cancer survivors (M = 31.9 ± 1.9 °C) was found to be moderately lower (*p* = 0.015, d = −0.615) than those of a group of women without the disease (M = 33.1 ± 0.69 °C) reported by Rodríguez [59]. However, when the post-mindfulness temperature was compared between the group of breast cancer survivors (33.3 ± 2.01 °C) and the disease-free group (33.8 ± 0.92 °C), no differences were found (*p* = 0.263). Neither were thermal differences found pre-compassion between cancer survivor women (33.5 ± 0.90 °C) and women without the disease (33.5 ± 0.70 °C, *p* = 0.858), nor post-compassion (33.9 ± 0.80 °C and 34 ± 0.72 °C, respectively) (*p* = 0.603).

In summary, the results suggest that female cancer survivors, when faced with a new situation (individual or group), have lower skin temperatures at rest than women without the disease; that is, they present greater sympathetic activation. However, once they become familiar with mindfulness exercises, they increase their skin temperature as much as that of a non-clinical group.

### 3.6. Thermal Image

**Figure 6:**
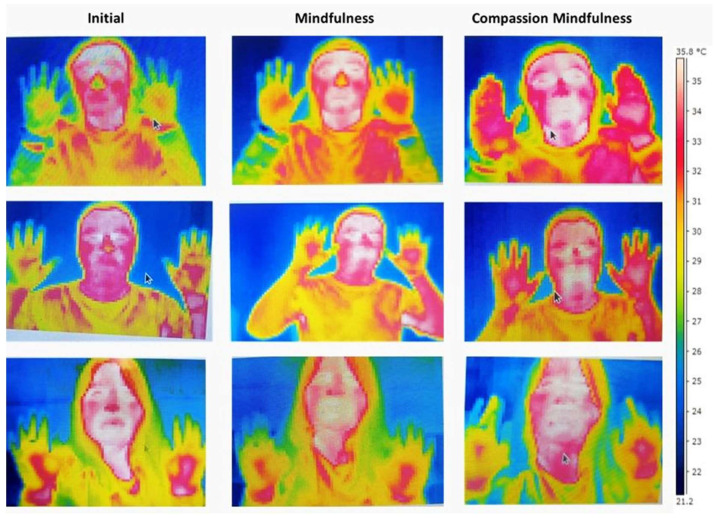
Infrared thermal imaging of resting (initial) state, mindfulness practice, and compassionate mindfulness.

To illustrate the psychophysiological effects of mindfulness and compassionate mindfulness, the thermal images shown in Figure 6 were taken, in which temperature elevations of the hands are identified, particularly the tip of the left middle finger, as well as the tip of the nose and the perioral region, including the lips.

## 4. Discussion

The interest of the present study was to examine the behavior of skin temperature (on the hands, in the context of the use of face masks due to the COVID-19 pandemic) in a single session of mindfulness and compassion with short-duration exercises in breast cancer survivors. Based on the objectives set, the mood, the capacity for full attention, compassion, the willingness to give and receive social support, and the perception of stress were evaluated, and the effect of a single session of full attention and compassionate full attention was examined on the temperature of the skin on the hands, an area sensitive to the affective psychophysiological response in a group of female survivors of breast cancer, applied both individually and in a group.

The results of the present study suggest: first, this group of women maintains a positive affective balance, with levels of mindfulness, compassion, and willingness to give and receive social support like non-clinical populations. These data are like those reported by Park, et al. [60], who suggest that breast cancer survivors without another disease have greater psychosocial health than women without an oncological diagnosis. Furthermore, significant direct associations were found between several positive affections, as well as between negative affects; meanwhile, inverse associations were presented between some positive affects with some negative ones, highlighting the significant inverse associations of feelings of loneliness and misery. Sharing feelings with other people was positively associated with the level of happiness. That is, expressing affection was related to the degree of well-being, as Cerezo, Ortíz & Cardenal [61] had suggested.

Second, the crossover repeated measures design managed to control the carryover effect of the stimuli, so a main effect was found only with the practice of mindfulness and compassionate mindfulness increasing skin temperature while no differences were recorded between remaining at rest and personal relaxation. Previous studies have reported the association between (perioral) skin temperature and positive feelings of social connection [62], where the increase has been linked to a state of calm and social involvement [63].

Third, the practice of mindfulness and compassionate mindfulness induced three effects: on the one hand, both procedures increased skin temperature (an indicator of the decrease in sympathetic nervous activity associated with stress), whereas compassion presented a size of greater effect. Furthermore, the subjective perception of stress was reduced. Finally, compassionate mindfulness promotes the experience of positive affections, mainly tranquility, relaxation, peace, and gratitude. These results are congruent with similar studies where the practice of compassion promotes subjective well-being and social benefits (promoting prosocial behaviors and sharing positive affect) in women with breast cancer [8,10,11,12,13,14,15,16,17,18,19,20].

According to the existing evidence, women with breast cancer can benefit positively from eight-session mindfulness programs; however, those who practice a single mindfulness session of between 5–30 min also present an immediate effect in reducing psychological distress and obtaining a relaxing and calming effect, in addition to reducing their suffering [41,42]; however, the results of the present study indicate positive physiological changes in the first 3 minutes of mindfulness and self-compassionate mindfulness-based practice, which provides relevant, although not conclusive, information regarding the duration of the techniques that may be useful in public hospital care services in Mexico, whose care has a limited duration.

These psychophysiological effects (autonomic and subjective) coincide with the evidence of psychosocial interventions to reduce biomarkers of chronic stress in patients with breast cancer reviewed by Mészáros, et al. [64], where it is suggested that the practice of mindfulness and breathing regulates neuroendocrine and immune activity, in addition to improving mood.

Among the limitations of the present study, firstly, due to the type of sampling, the lack of external validity that allows drawing generalizable conclusions to groups with the same diagnosis stands out. This group has a high degree of social cohesion, and most of its members have learned to live with cancer and its oncological treatment due to the regularity of their group meetings with oncology and other health services. Second, the N of the study is low, and it is not intended to generalize the results found; that is, the results found must be considered for this specific group and at the time of the circumstances in which the effects of the intervention were examined. Personal relaxation is the ability to calm down on your own without the support of a psychologist. The selected design allows us to evaluate whether personal relaxation alone could increase skin temperature, or whether it is attributable to the practice of mindfulness. However, the nature of the research design limits examining the temporality of the psychophysiological effects achieved with mindfulness practice.

It is recommended to provide regular follow-up with a mindfulness program to this group, to examine not only the immediate psychophysiological effect of the single-session practice but also its biopsychosocial scope in the medium and long term.

In mental health programs, psychosocial intervention can reduce distress. However, not all psychological techniques are effective for all patients, and a non-invasive objective measure to examine the autonomic response associated with stress is the psychophysiological recording of skin temperature using infrared sensors.

Some people are more susceptible than others to achieving a level of subjective well-being through mindfulness, so it is necessary to evaluate well-being and the capacity for mindfulness both pre-, post-, as well as its long-term effects. Psychometric measurement is quick and useful for assessing affect. For its part, the psychophysiological record allows us to identify patients with low, moderate, or high physiological tension associated with stress.

Public mental health programs can expand their clinical criteria with a multiaxial evaluation, which considers the clinical observation of signs and symptoms, psychometric measurement, and psychophysiological evaluation,

However, we do not intend to generalize the results but rather to examine whether the practice of mindfulness and compassion promotes positive affect and reduces sympathetic tone by increasing temperature, achieving a subjective state of tranquility.

These results should be considered with caution since this group of cancer survivors was already formed previously. They were already known to one another, and therefore an interaction effect is possible between the familiarity of each patient with her colleagues and the effect of the treatment. It can be replicated in a newly formed group. Those who are new to practicing brief mindfulness and compassion exercises can familiarize themselves with online techniques of professional mental health instruction from their authors, Erick López-Maya (in Spanish) and Kristin Neff (in English) [65,66].

## 5. Conclusions

Mindfulness and compassion programs have been recommended as a practice to improve mental health in different groups vulnerable to chronic stress. The results found from single-session practice, both individually and in groups, provide evidence about skin temperature as an autonomic biomarker of the affective state induced by mindfulness and compassion. Furthermore, portable infrared thermometers are a viable low-cost option for psychosocial research: the temperature increase only occurs when mindfulness or compassionate mindfulness exercise are applied, while self-relaxation is no different from staying in repose.

## Correction Statement

Following the removal of this article from the Special Issue “Thermology Health Care Applications and Medical Thermography”, an Editorial Board member reviewed the entire editorial process and confirmed all elements had been carried out as per MDPI Editorial Process policy (https://www.mdpi.com/editorial_process). As a result, the original Academic Editor listed on this publication has been replaced with that of the Editorial Board member who conducted this review. The original publication has now been updated accordingly. This information has also been transmitted to relevant indexing organizations.

## Appendix A

The Self-Compassion mindfulness exercise consisted of the following meditation:

1. *Breathe deeply and close your eyes.*
2. *Recognize how things are for you at this moment, and when you are ready, begin to become aware of your body.*
3. *Bring to mind an image of a person you love, someone who easily evokes feelings of warmth and love.*
4. *Imagine telling them: may you be happy; may your mind and body be healthy. May you feel protected from all internal and external damage. May you have love and affection. May you experience inner peace.*
5. *Take a moment to feel what it’s like to want these things for him or her. Project these feelings onto yourself, letting the feelings of love and security fill you, knowing that there is nothing you can do to deserve these feelings and desires, which are given freely, without conditions.*
6. *Become aware that this is what you wish for yourself, and mentally repeat the following phrases: may I be happy, may my mind and body be healthy. May I feel safe and protected from all internal and external harm. May you have love and affection. May you experience inner peace.*
7. *Give yourself a few moments to return, feel yourself here, in this moment and when you feel ready, open your eyes.*

## Appendix B

The breathing exercise with mindfulness consisted of the following guided meditation:

1. *I invite you to sit with your feet firmly on the ground and your back straight; close your eyes.*
2. *Bring your attention to your breathing and notice it with curiosity, as if you have never paid attention to your breathing.*
3. *Notice the air, entering through your nostrils and going down to the bottom of your lungs, and notice how it flows.*
4. *Notice the air entering and leaving your nostrils, notice how it is a little warmer as it leaves, and a little cooler as it enters.*
5. *Notice the subtle rise and fall of your shoulders, the gentle rise and fall of your chest, and the relaxing expansion and contraction of your abdomen.*
6. *Pay attention to one of these areas, whichever you prefer. The air enters and leaves the nasal passages. To the rise and fall of your chest. Or the movement of your abdomen.*
7. *Keep your attention there, noticing the movement in and out as you breathe.*
8. *Any feelings, impulses, or sensations that arise, whether pleasant or unpleasant, acknowledge them gently as if you were nodding your head to people passing by you on the street.*
9. *Gently acknowledge their presence and let them be. Allow them to come and go as they please.*
10. *Whatever thoughts, images, or memories arise, whether comfortable or uncomfortable, simply acknowledge them and allow them to be.*
11. *Let them come and go as they please and return your attention to your breathing.*
12. *From time to time your attention will wander as you get caught up in your thoughts, each time this happens, notice what distracted you and then bring your attention back to the breath.*
13. *No matter how often you stray, whether a hundred times or a thousand, your goal is simply to notice what distracted you and refocus on your breathing.*
14. *If frustration, boredom, anxiety, impatience, or other feelings arise. Simply acknowledge them and keep your attention on your breathing.*
15. *When you are ready, bring your attention back to the room. The time has come to return, you breathe deeply, inhale, and exhale. Little by little move your body, little by little move your hands, little by little, and gently open your eyes, to your time and your space.*
16. *Give yourself a few moments to return, move your body, and feel yourself here, in this moment.*

## Appendix C

STOP: Stop, Take a breath, Observe, and Proceed, whose script was the following:

1. *Stop: Pause what you are doing or thinking. This is done to realize whether you are acting automatically.*
2. *Take a breath: Deep breathing focuses you on yourself and the present moment.*
3. *Observe your body: the physical sensations you are aware of, touch, sight, sounds, smells, and tastes, your emotions (what are you feeling right now?), and your thoughts (what interpretations are you making of your feelings and emotions?).*
4. *Proceed: continue with what you interrupted, making a conscious effort to incorporate what you just used.*

## Appendix D

Compassionate mindfulness exercise (Compassionate Breathing & Feeling Safe with Your Benefactor), the script of which was the next:

1. *Sit upright feel your feet firmly on the floor and cross your hands in front of your heart.*
2. *Visualize yourself as an innocent child or, if it is easier for you, any innocent child.*
3. *Be aware of your breathing. As you inhale, notice how your breath moves toward your chest and lower toward your abdomen. Then there is a small natural pause before releasing the air that rises upward and passes through the nostrils. However, if you have a cold, or for any other reason you must breathe through your mouth, open it slightly, and breathe gently. On the exhale, imagine that you are blowing on a spoonful of hot soup, and you don’t want to spill it; this will simulate nasal breathing. Continue to focus on your natural breathing for a while until you feel more and more comfortable.*
4. *Now start focusing on “the little child in your heart”. What would you like to say? Let compassionate, warm words and phrases arise in your mind as if you were trying to calm an angry child. I prefer not to say what language you should use. I just hope you can stay in your heart and let kind words emerge.*
5. *After a while, return to your breathing. Little by little, put your hands in your lap and continue breathing for a while longer.*
6. *Visualize yourself in your favorite safe place, such as a meadow, a room, or a garden.*
7. *Now watch as the person who creates a sense of security in you walks towards you with open arms. When they arrive, they hug you or hold your hands. Internally, listen to them say the words “security”, “protection”, and “peace”; or if you prefer, let them talk or sing you a relaxing lullaby.*
8. *Place your hand in the center of your heart and stay with this visualization for as long as you wish.*
